# A 5‐month‐old infant with a pineal tumor

**DOI:** 10.1111/bpa.70066

**Published:** 2025-12-29

**Authors:** Fabien Forest, Martin Hasselblatt, Fanélie Barral‐Clavel, Alix Clemenson, Sandrine Thouvenin, Claire Berger, Anne Mc Leer, Catherine Godfraind

**Affiliations:** ^1^ Department of Pathology and Molecular Pathology University Hospital of Saint Etienne Saint Etienne, Cedex 2 France; ^2^ Institute of Neuropathology University Hospital Münster Münster Germany; ^3^ Department of Neurosurgery University Hospital of Saint Etienne Saint Etienne, Cedex 2 France; ^4^ Department of Pediatric Oncology University Hospital of Saint Etienne Saint Etienne, Cedex 2 France; ^5^ Department of Biopathology Centre Léon Bérard Lyon France; ^6^ Neuropathology Unit, Clermont‐Ferrand University Hospital Clermont‐Ferrand France

BOX 1 **Virtual glass slide**

Access at https://isn‐slidearchive.org/?col=ISN&fol=Archive&file=BPA‐25‐02‐CIR‐062.svs.

## 1 CLINICAL HISTORY

**FIGURE 1:**
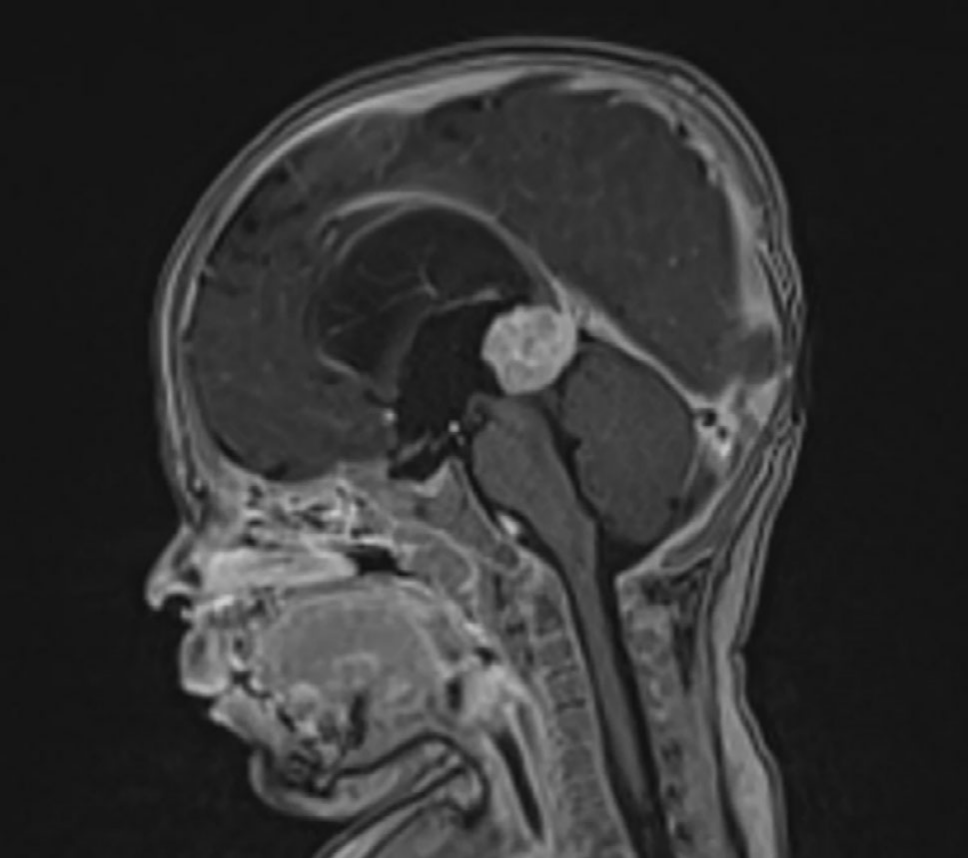
MRI, sagittal plane, T1 sequence after gadolinium injection.

A 4‐month‐old child was referred to our institution due to partial epilepsy accompanied by asthenia, downward gaze deviation, and strabismus. Brain magnetic resonance imaging (MRI) showed a well delineated tumor of the pineal region in the posterior part of the third ventricle with acute hydrocephalus. The tumor was in isosignal in T1 and T2 with contrast enhancement after gadolinium injection measuring 18 mm (Figure 1). A surgical excision was decided through suboccipital transtentorial approach; during surgery, the tumor could not be completely removed because of venous invasion and because of a huge Galen vein preventing access to part of the tumor.

## 2 FINDINGS

**FIGURE 2:**
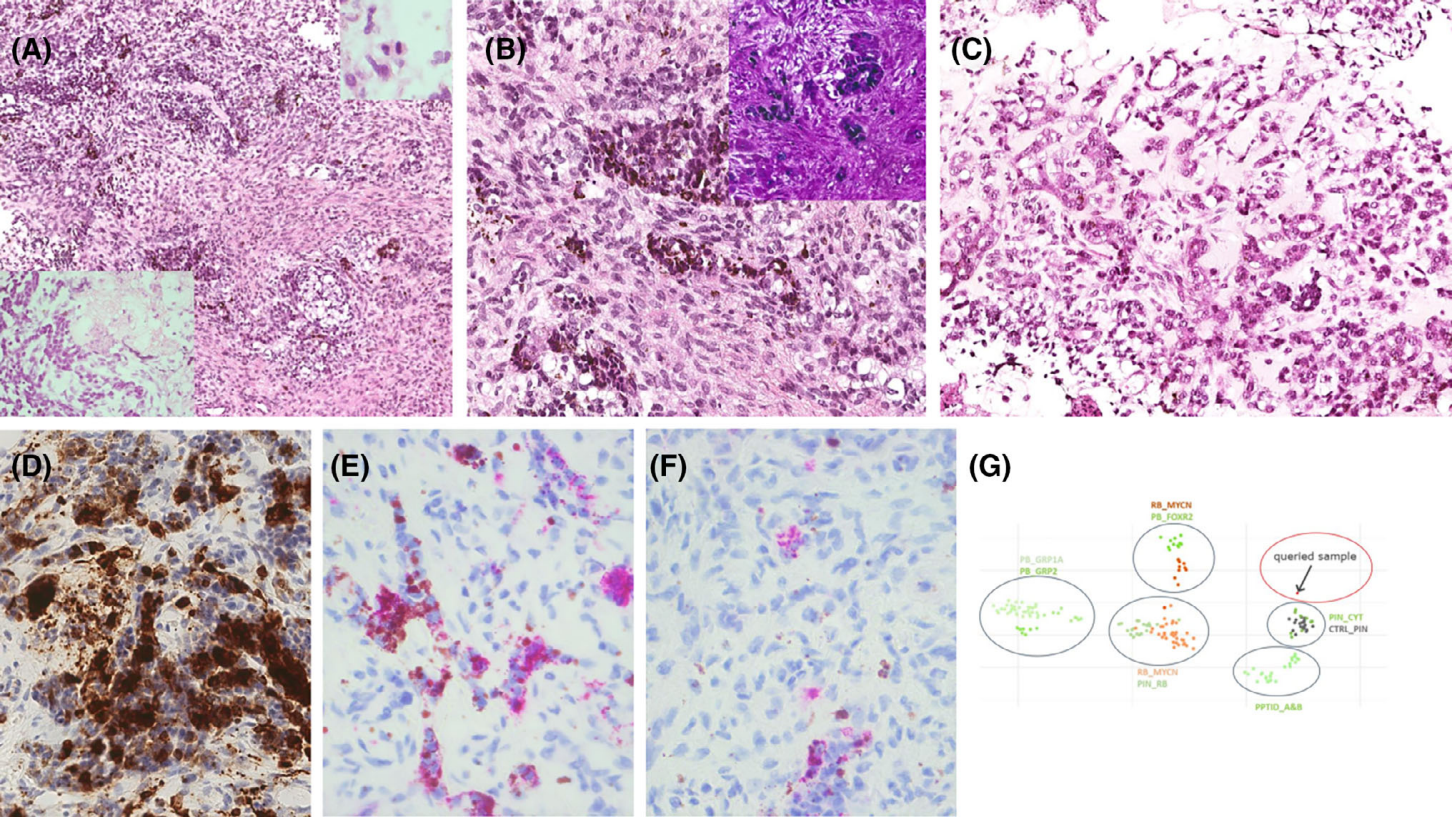
(A) HE ×100, upper right inset ×400 mitoses, lower left inset: necrosis. (B) HE ×200, inset Fontana Masson stain. (C) Cartilage‐like stroma. (D) Synaptophysin stain, ×400. (E) HMB45 stain, ×400. (F) Melan‐A stain, ×400. (G) t‐SNE analysis.

Histopathological analysis revealed a highly cellular tumor composed of sheets and trabecular structures made of cells with hyperchromatic nuclei and high nuclear cytoplasmic ratio, with numerous mitoses and areas of necrosis (Figure 2A and Box 1). Tubular structures containing melanin pigment were observed, as confirmed by Fontana‐Masson staining (Figure 2B inset). No ganglion cell, nor rhabdomyoblastic cells, nor cells with striated muscle differentiation were found after examination, but cartilaginous‐like hyalinized stroma areas were found (Figure 2C). Tumor cells stained positive for synaptophysin (Figure 1D). Expression of SMARCB1/INI1 and SMARCA4/BRG1 was retained. Stain for Lin28 was negative, while stains for Melan‐A and HMB45 were weak and focal (Figure 2E,F). The Ki67/MIB1 proliferation index was 70%. Methylation testing on formalin‐fixed paraffin‐embedded tissue, on a tumor with tumor purity at 80% and 500 ng of DNA input with Infinium Methylation EPIC v2.0. The DNA methylation profile of the tumor did not group with any of the DKFZ classifier (version 12.8). The NIH classifier grouped the tumor with neuroblastic embryonal tumors with a score at 0.976 without a sufficient score to assess a specific tumor type. On t‐SNE analysis, the DNA methylation profile grouped in the vicinity of other pineal tumors and normal pineal gland (Figure 2G).

## 3 DIAGNOSIS

Pineal anlage tumor, CNS WHO grade 4.

## 4 DISCUSSION

Pineal anlage tumor is regarded as a morphological variant of pineoblastoma. The histopathological hallmarks are the presence of a neuroectodermal component constituted of pineoblastoma‐like cells with neuronal, glial, or melanin‐containing cells combined to an ectomesenchymal component containing rhabdomyoblasts, cartilage, or smooth muscle. In the English literature, less than 30 cases have been reported [1]. Histopathological examination is not specific, with embryonal‐like neoplastic cells growing as sheets or trabeculae, but the information of the location of the tumor is important for the diagnosis. The main differential diagnosis is atypical teratoid/rhabdoid tumors and embryonal tumor with multilayered rosettes. Pineoblastomas, so as pineal anlage tumor, retain expression of SMARCB1(INI1) and SMARCA4 (BRG1) and do not express LIN28A.

The molecular alterations of pineal anlage tumors have rarely been documented because of their rarity. Nevertheless, none of the tested pineal anlage tumor demonstrated the characteristic alterations of the four molecular subgroups of pineoblastoma. Methylation profiles are also scarce. Strikingly, pineal anlage tumors do not group with pineoblastoma [1]. However, epigenetic similarity with medulloblastoma group 3 has been described, similarly to melanotic neuroectodermal tumor of infancy [2]. In contrast to pineal anlage tumors, however, the latter typically follows a relatively benign course. Exploratory tSNE based on few cases demonstrates that two of the pineal anlage tumors grouped with pineoblastoma MYC/FOXR2 [1, 3]. Additional cases are needed to further characterize pineal anlage tumors and to understand if this tumor represents a morphological variant of pineoblastoma or a distinct tumor type that may be a pineoblastoma subtype with specific molecular alterations or an independent entity.

## AUTHOR CONTRIBUTIONS

**FF:** Writing; data collection; review. **MH:** Reviewing of the slides; critical review of the manuscript. **FBC:** Data collection; follow‐up; critical review of the manuscript. **AC:** Data collection; critical review of the manuscript. **CB:** Data collection; follow‐up; critical review of the manuscript. **AML:** Molecular analysis; critical review of the manuscript. **CG:** Data collection; follow‐up; critical review of the manuscript; conceptualization. FF is responsible for the accuracy of the presented data.

## CONFLICT OF INTEREST STATEMENT

The authors declare no conflicts of interest.

## ETHICS STATEMENT

All data related to this case are deidentified.

## Data Availability

The data that support the findings of this study are available from the corresponding author upon reasonable request.
